# Supplementary material for: A strong fracture-resistant high-entropy alloy with nano-bridged honeycomb microstructure intrinsically toughened by 3D-printing
Source: Nat Commun. 2024 Jan 29;15:841. doi: 10.1038/s41467-024-45178-2 (PMC10825177; doi:10.1038/s41467-024-45178-2)
Supplement: Supplementary file 1 — Supplementary Material [file 41467_2024_45178_MOESM1_ESM.pdf]

Supplementary Information

A strong fracture-resistant high-entropy alloy with nano-bridged honeycomb microstructure intrinsically toughened by 3D-printing

Punit Kumar[1,2*], Sheng Huang [3*], David H. Cook[1,2*], Kai Chen[4], Upadrasta Ramamurty[3,5], Xipeng Tan[6#], Robert O. Ritchie[1,2#]

[1]Department of Materials Science and Engineering, University of California, Berkeley, CA, USA

[2]Materials Sciences Division, Lawrence Berkeley National Laboratory, Berkeley, CA, USA

[3]School of Mechanical and Aerospace Engineering, Nanyang Technological University, Singapore

[4]Center for Advancing Materials Performance from the Nanoscale (CAMP-Nano), State Key Laboratory for Mechanical Behavior of Materials, Xi'an Jiaotong University, China

[5]Institute of Materials Research and Engineering, Agency for Science, Technology and Research (A*STAR), Singapore

[6]Department of Mechanical Engineering, National University of Singapore, Singapore

[*]Authors with equal contribution; [#]Corresponding authors

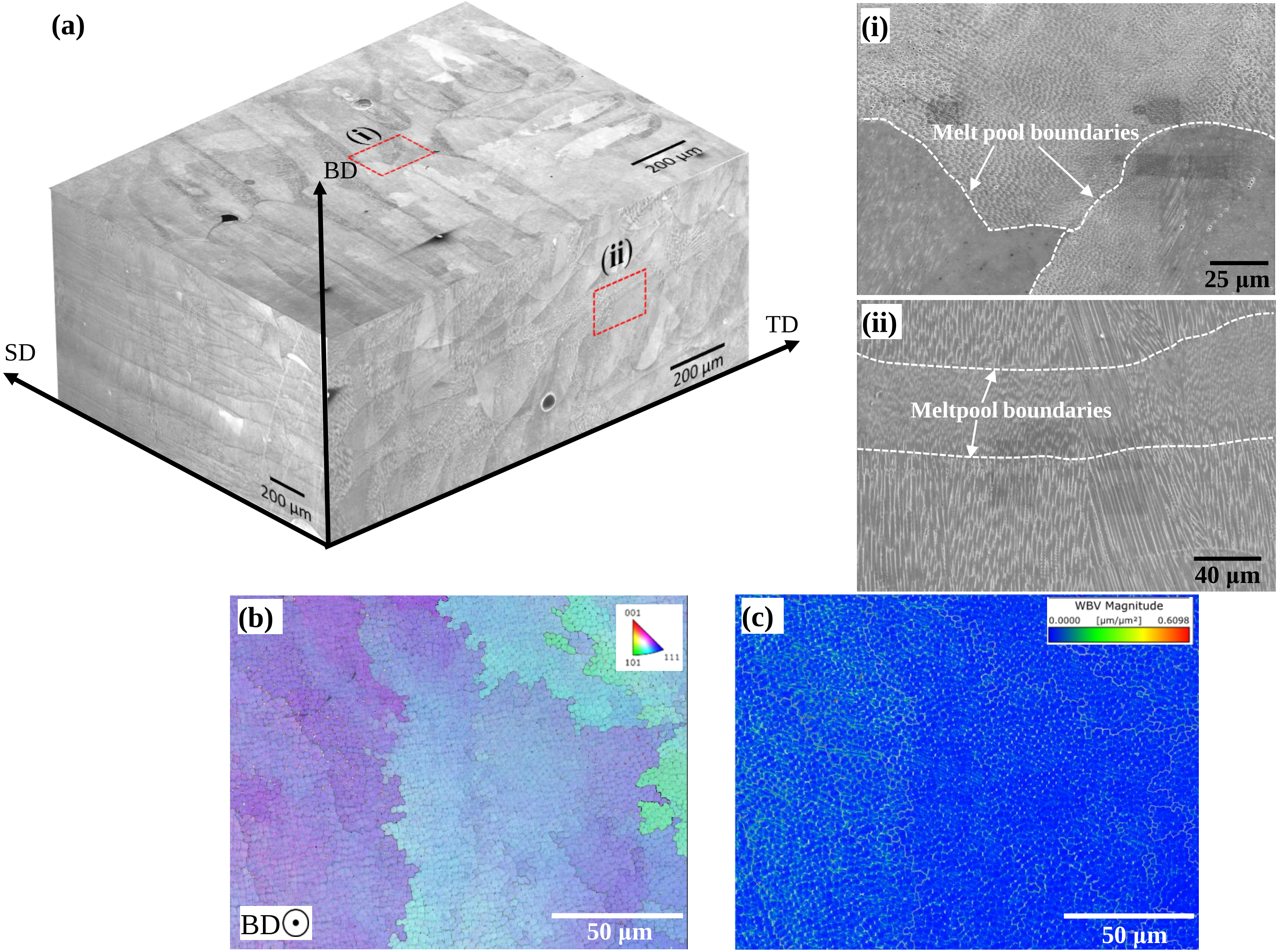

Supplementary Figure 1. (a) Representative 3D microstructure showing laser scan tracks and/or melt pool boundaries on the planes parallel and perpendicular to the build direction, BD. The magnified images from locations (i) and (ii) show meltpool boundaries. (b) EBSD inverse pole figure (IPF) map shows the texture and misorientation distribution across the grains and the cellular structure. (c) The corresponding weighted Burgers vector map of the IPF map show dislocation distribution in as-built conditions.

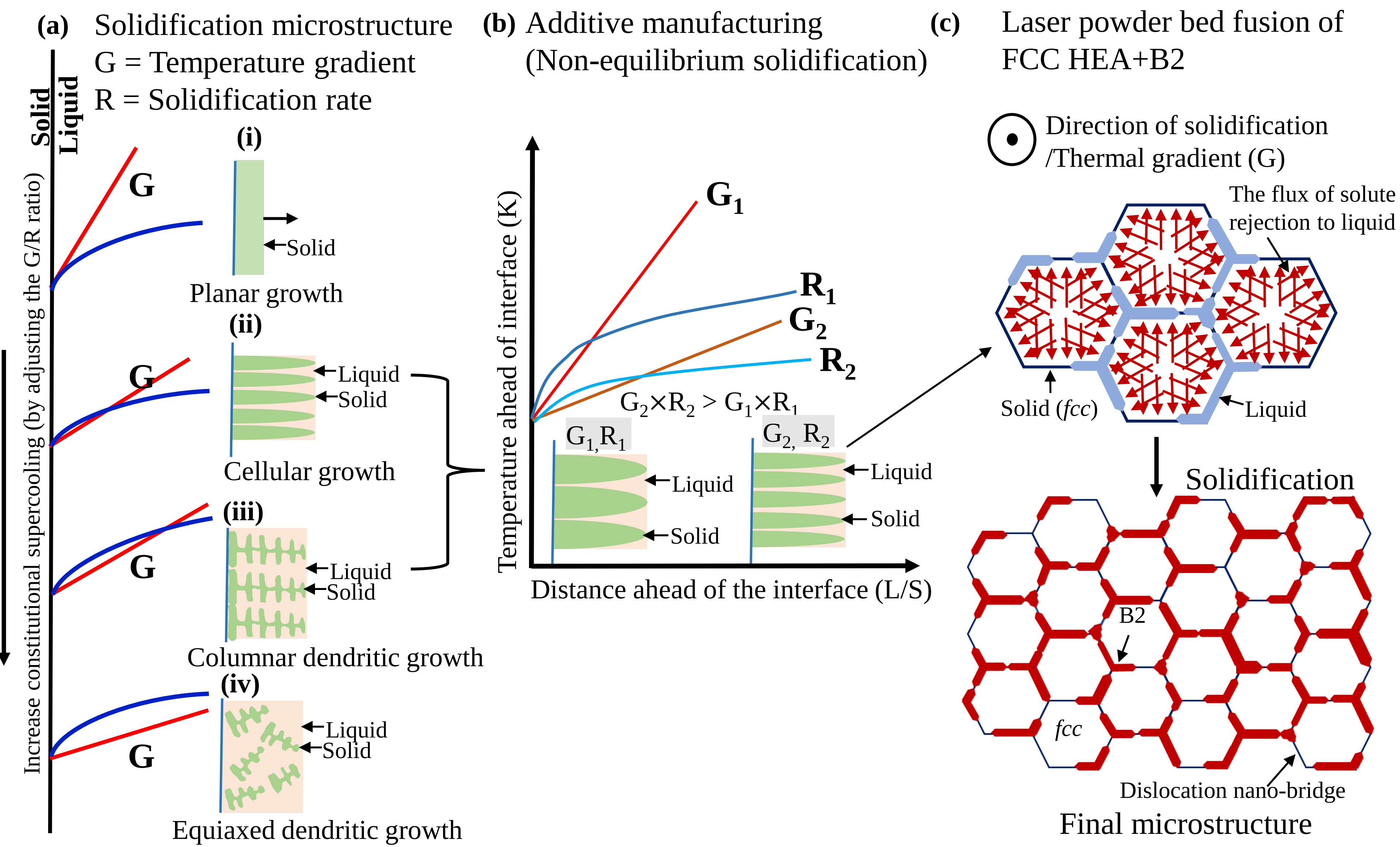

Supplementary Figure 2. (a) Schematic showing the effect of constitutional supercooling on the solidification modes, (i) planar; (ii) cellular; (iii) columnar dendritic, and (iv) equiaxed dendritic. (b) Schematic showing the effect of thermal gradient (G) and solidification rate (R) on the morphology of the cellular structure during non-equilibrium rapid solidification. (c) Schematics showing microstructural evolution in $Al_{0.5}CoCrFeNi$ produced by laser powder bed fusion (L-PBF) process. The cellular growth promotes solidification of the *fcc* phase (cells), resulting in the solute rejection to the liquid on the cell boundaries. The triple points of the cells see the highest flux of solute rejection. Therefore, the B2 phase forms primarily on the triple points of the cell boundaries. In the regions where Al is insufficient to form the B2 phase, the dislocation entangled there forms a nano-bridge connecting the micro-scale *fcc* cells (Figure 1D).

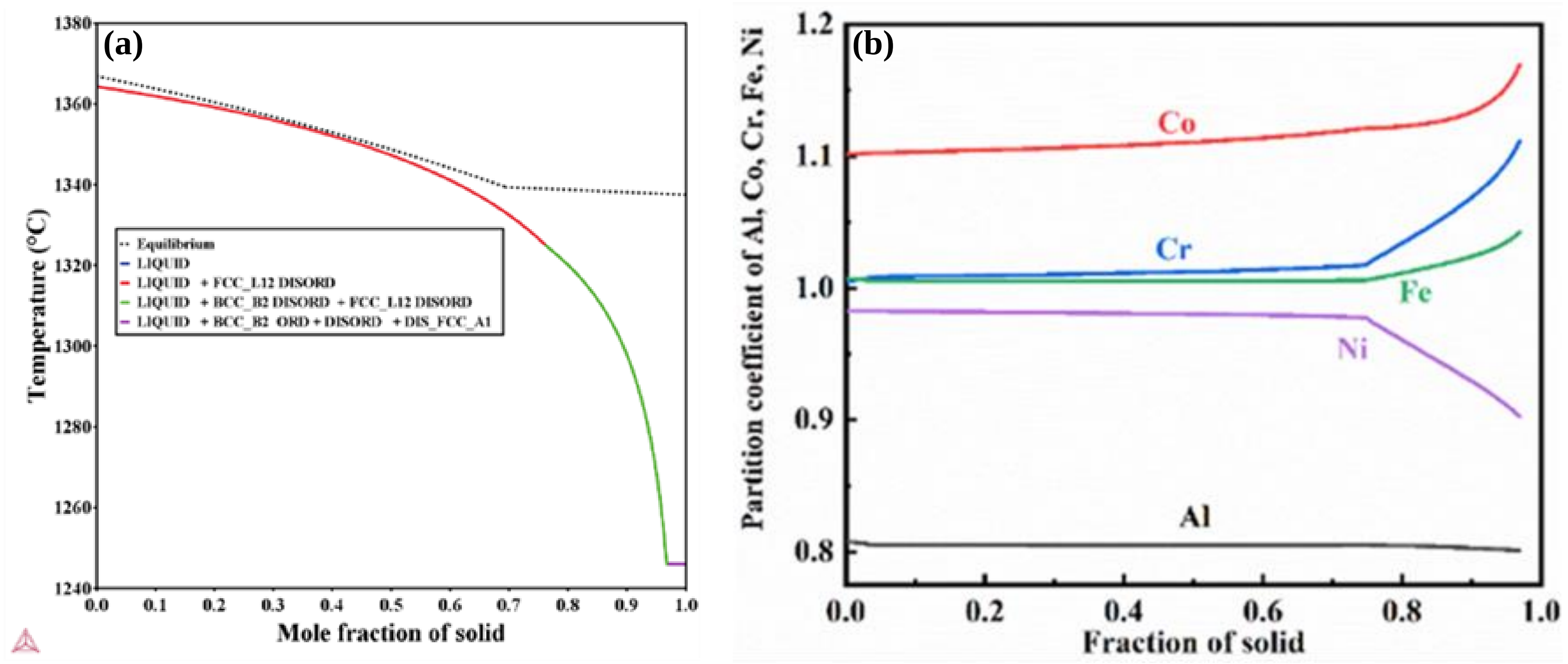

Supplementary Figure 3. (a) Solidification paths of $Al_{0.5}CrCoFeNi$ HEA and (b) equilibrium partition coefficients of the components Al, Cr, Co, Fe, and Ni in the HEA calculated by the Scheil-Gulliver model using Thermocalc software (TCHEA6 database).

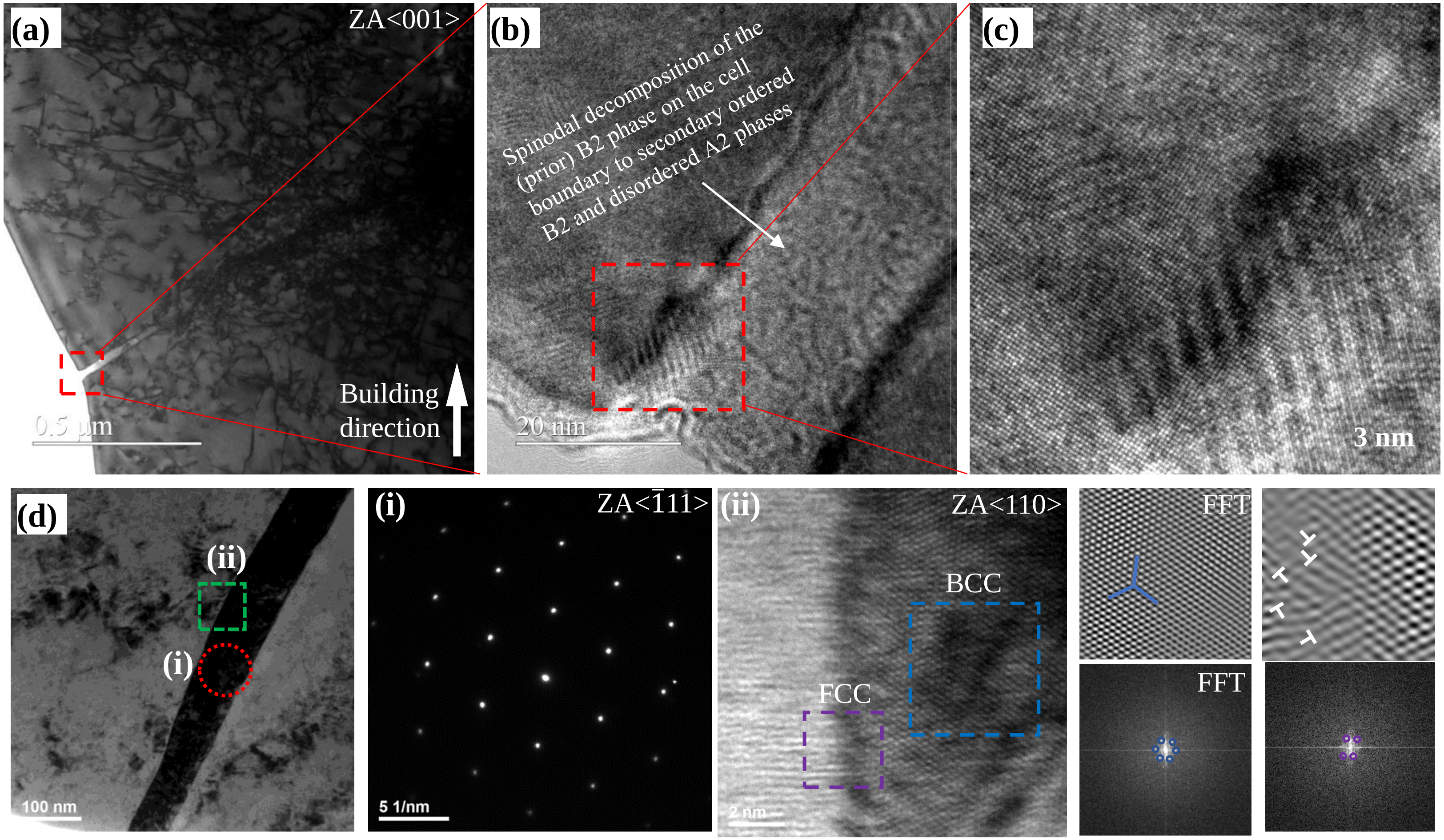

Supplementary Figure 4. (a) Transmission electron microscopy image of the (prior) B2 phase on the honeycomb cell boundary. (b) The (prior) B2 phase on the cell boundaries goes through spinodal decomposition during the LPBF process to form a mixture of *bcc* phases (secondary ordered B2 precipitates and disordered A2 precipitates). (c) The magnified image of the interface between the *fcc* matrix. (d) (i) TEM diffraction pattern of the B2 phase on the cell boundary, and (ii) Fourier transformed image showing the atomic distribution along the incoherent interface of *fcc* and *bcc* phases.

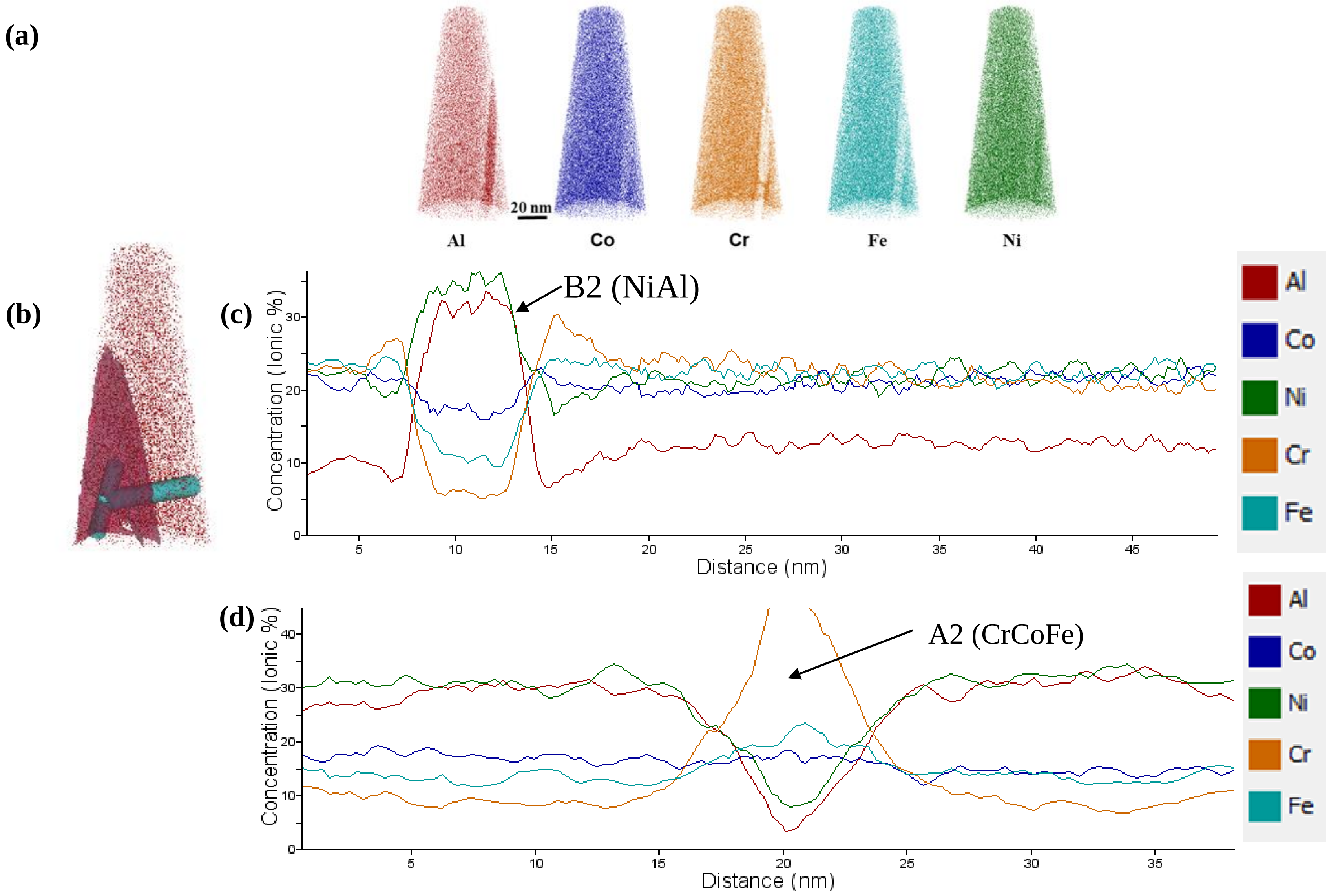

Supplementary Figure 5. (a) APT reconstructed volumes showing elemental distribution in both the *fcc* cell and intercellular B2 and the *bcc* phases, (b) Two region of interest cylinders are created to identify phases in the APT reconstructed volume in (a). 1D concentration profiles showing (c) distribution of different elements across the cell boundaries, where an intercellular secondary B2-NiAl phase is identified, and (d) evidence of spinodal decomposition of the intercellular phase primary B2 into secondary B2-NiAl and A2 (Cr-rich *bcc)* phases.

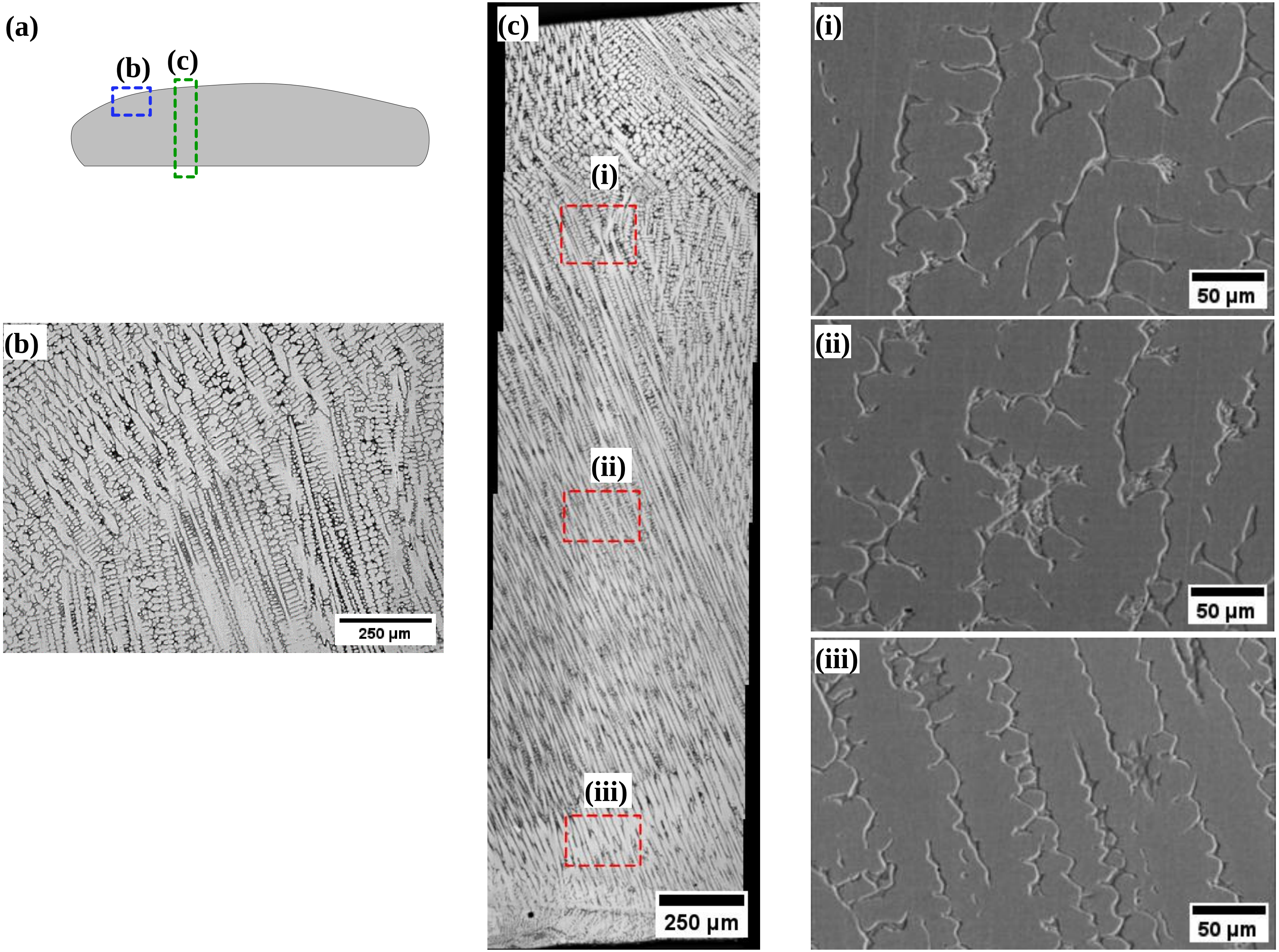

Supplementary Figure 6. (a) Schematic of arc melted $Al_{0.5}CoCrFeNi$ button ingot. (b) Magnified image showing the distribution of the B2 phase in the top region of the button. (c) microstructure variation across the height of the as-cast buttons. Magnified images from locations (i), (ii), and (iii) show the network of the B2 phase between the *fcc* dendrites. The thickness of the network of the B2 phase ranges between 2 - 30 µm at different locations.

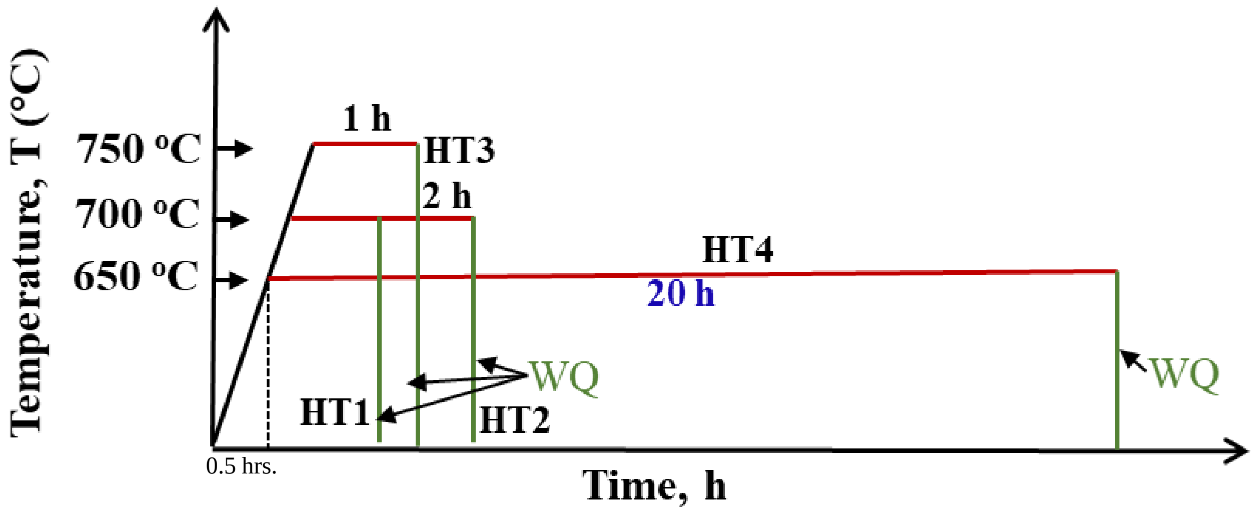

Supplementary Figure 7. Schematic illustration of the heat treatments employed on additively manufactured samples to optimize their tensile properties. Heat treatment schedule HT3 was employed on the C(T) specimen for fracture toughness evaluation. All the specimens were water quenched following the heat treatment.

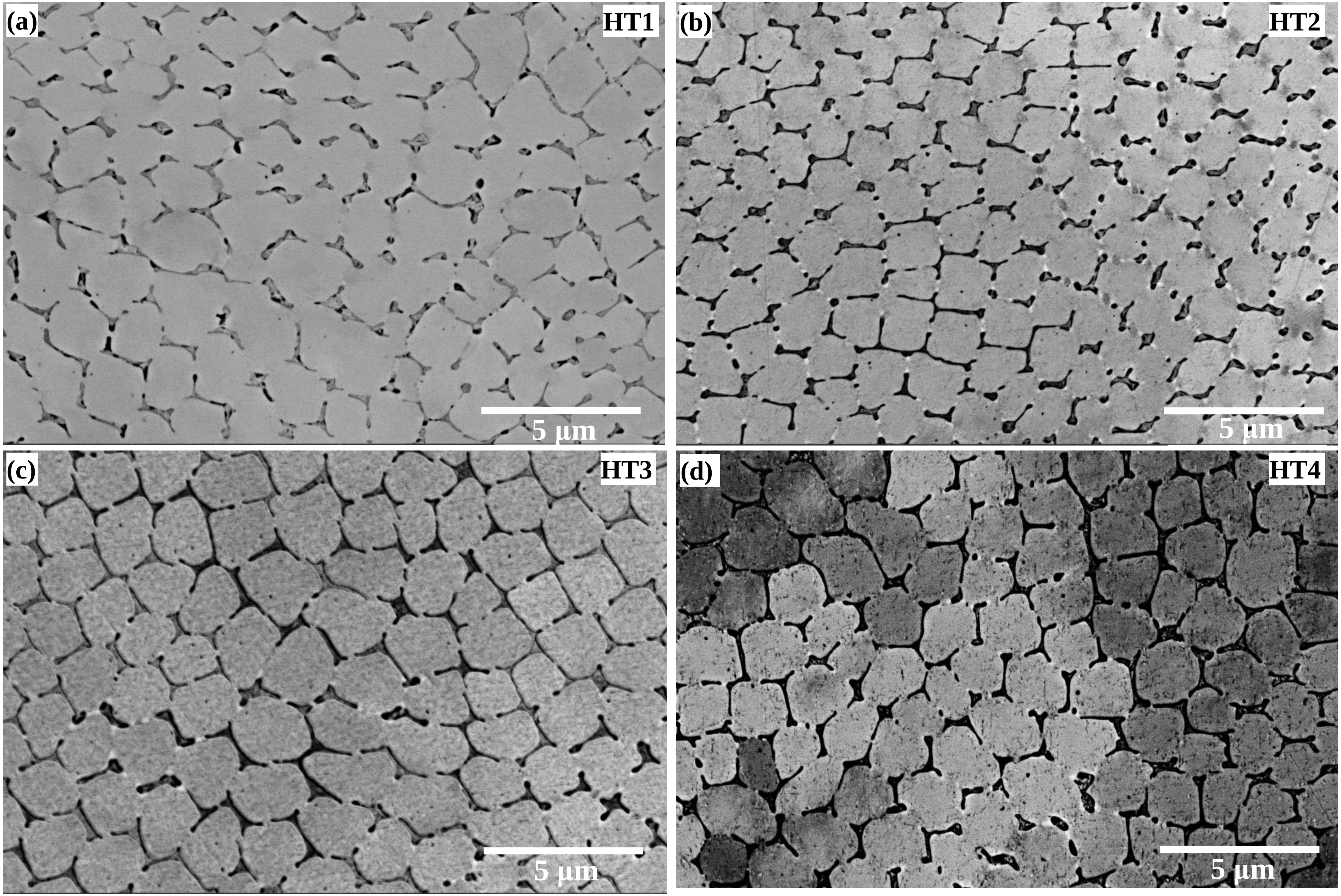

Supplementary Figure 8. Back-scattered electron images showing the morphology of nano-bridged cellular structures after heat treatments (a) HT1, (b) HT2, (c) HT3, and (d) HT4.

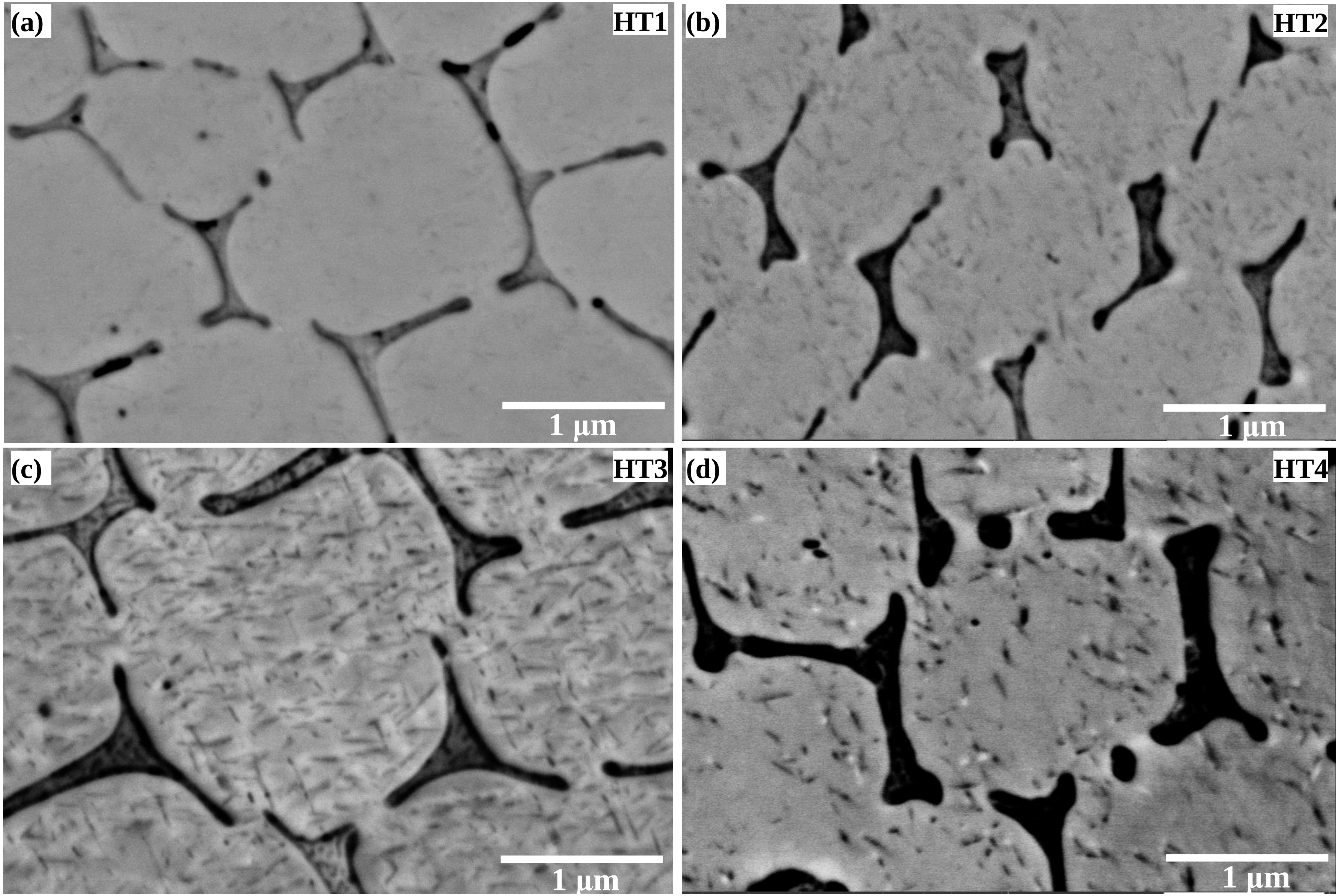

Supplementary Figure 9. High-resolution back-scattered electron images showing the morphology of B2 precipitates inside the cellular structures (*fcc* matrix) after the heat treatments (a) HT1, (b) HT2, (c) HT3, and (d) HT4.

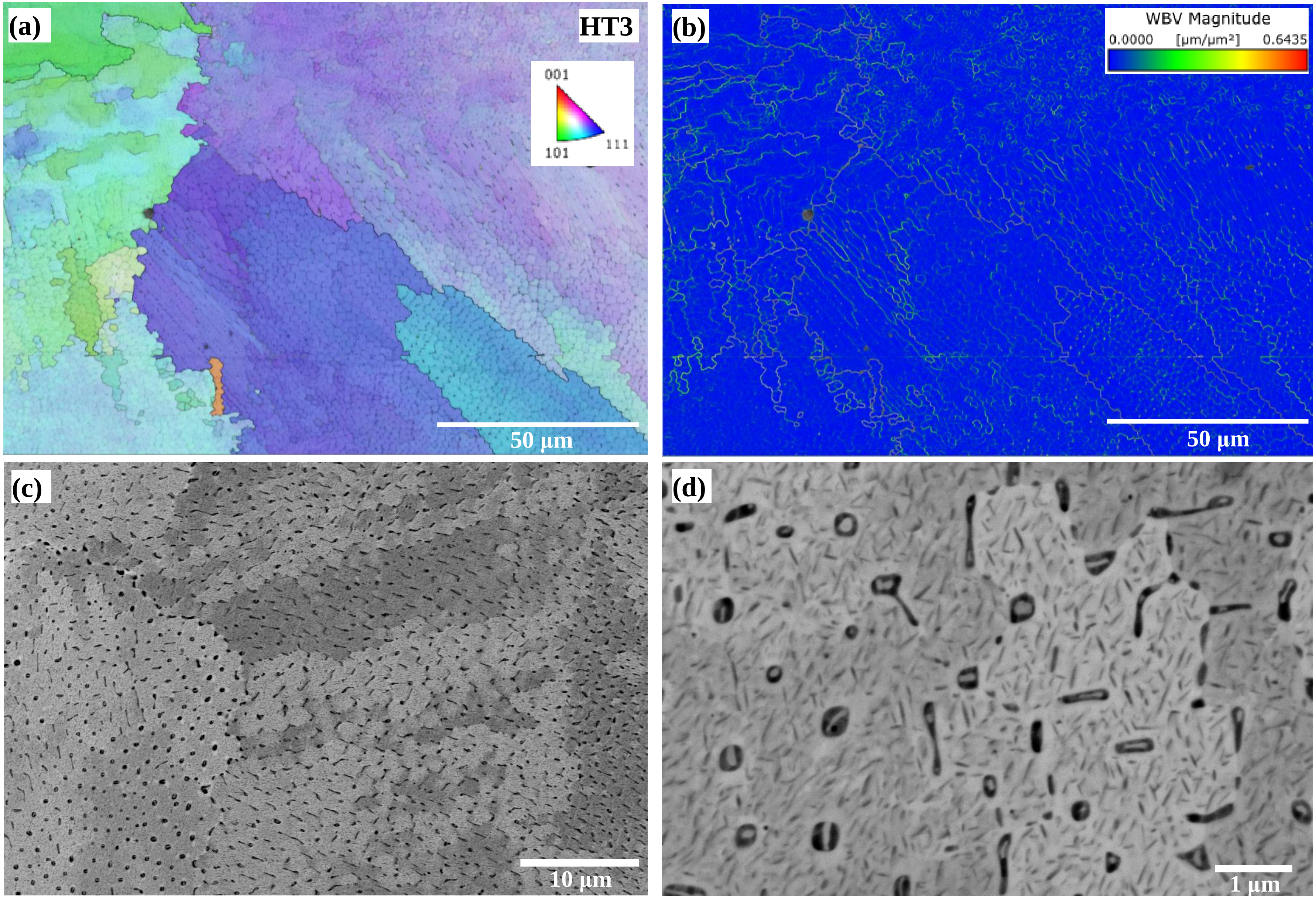

Supplementary Figure 10. (a) The EBSD inverse pole figure (IPF) map of the specimen subjected to heat-treatment HT3. (b) The corresponding weighted Burgers vector map shows dislocation distribution after the heat treatment. (c) An electron-backscattered contrast image obtained by a scanning electron microscope shows the microstructure after the heat treatment. (d) Nanoscale B2 precipitates are uniformly distributed in the microstructure after the heat treatment.

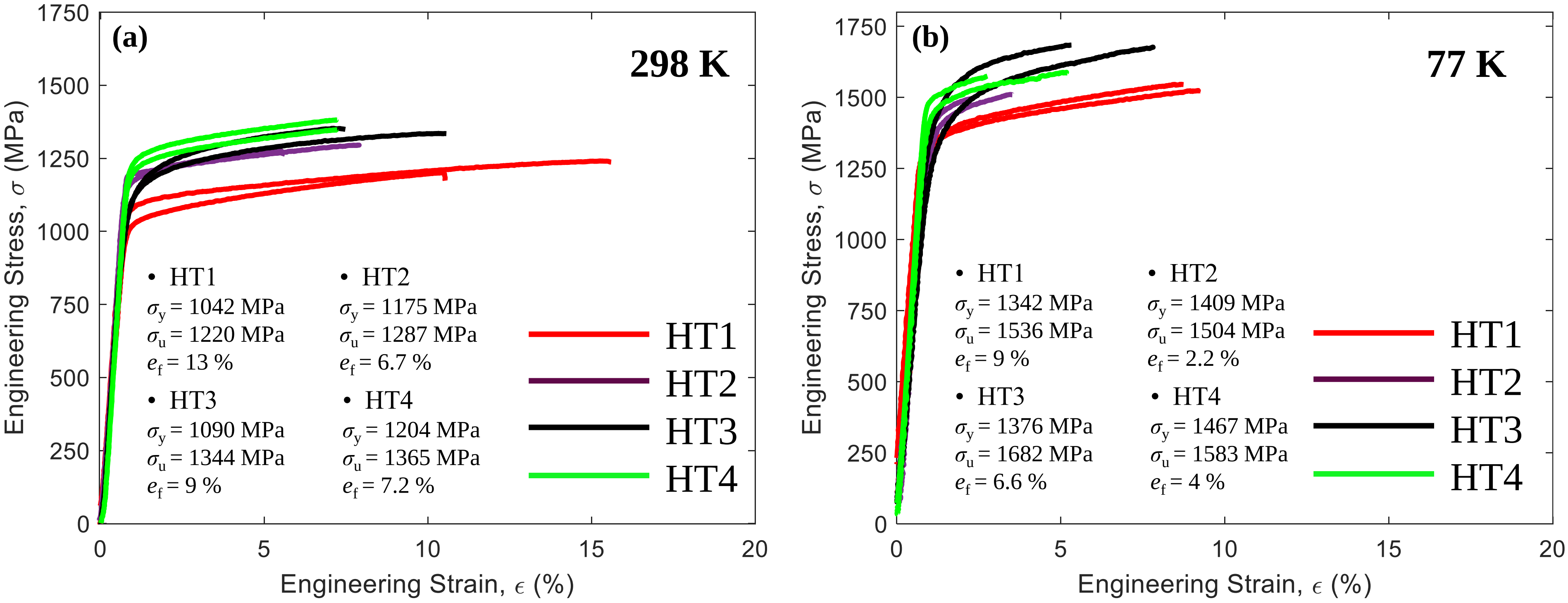

Supplementary Figure 11. Engineering stress, *S vs.* engineering strain, *e* curves of the various microstructures obtained by different heat treatments at (a) 298K, (b) 77 K. HT3 specimens show the best combination of strength and ductility at both these temperatures.

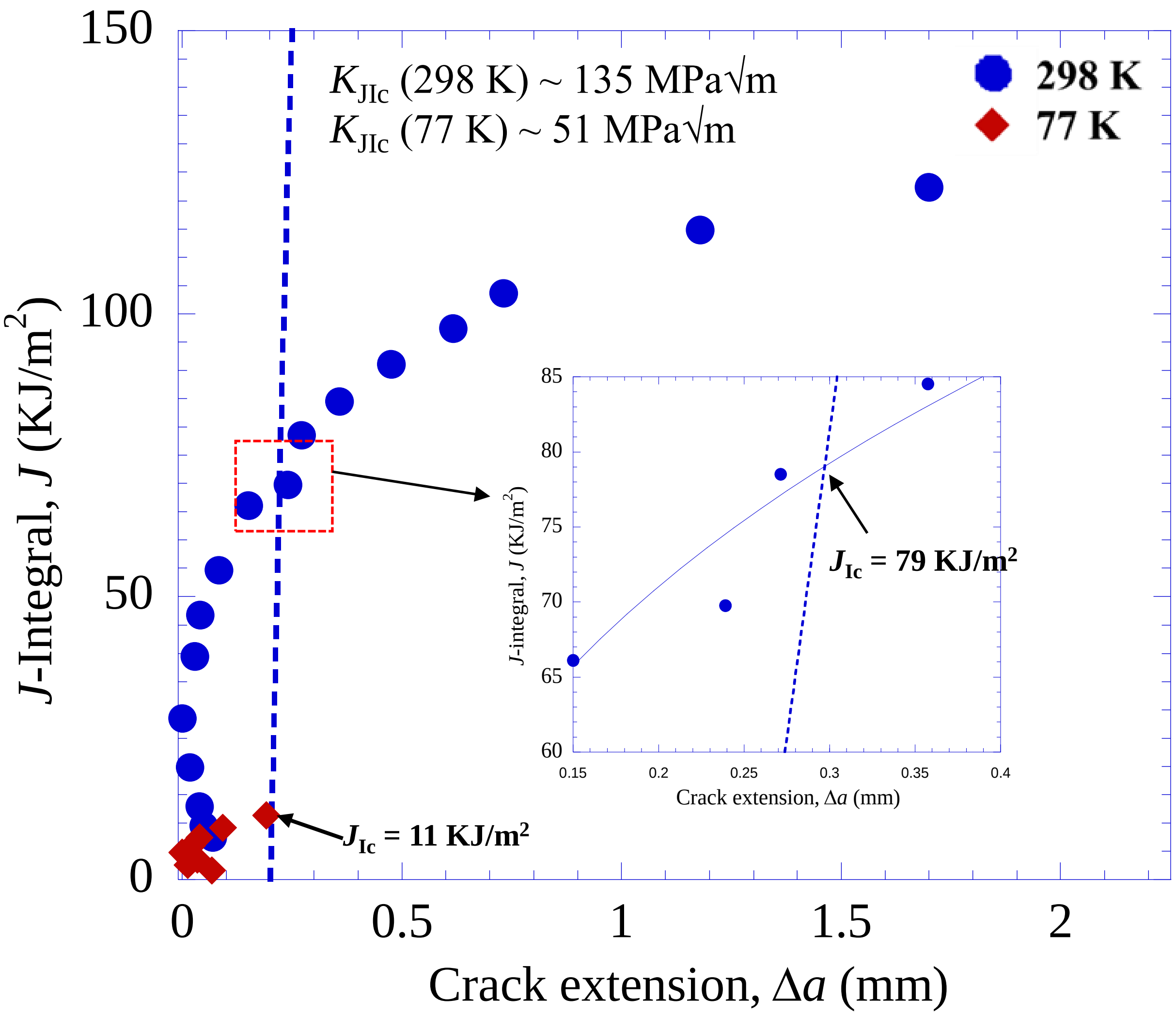

Supplementary Figure 12. Crack-resistance curves (*R*-curves) showing the crack-initiation fracture toughness, K_{JIc}, of the microstructure after the heat treatment at 750 °C for 1 hr (HT3). The K_{JIc} values are ~135 MPa√m and ~51 MPa√m at 298 K and 77 K, respectively. The negative crack extension in the initial few points of the *J*-*R* curve is a consequence of swayback caused by slippage between the pin and the C(T) sample/fixture at a relatively low applied load. After heat treatment, the HT3 sample becomes harder, yield strength (YS) approaching that of peak-aged maraging steel—the material of our pin—the indentation/brinelling of the pin onto the C(T) specimen hole becomes insufficient, leading to slippage at low force. However, as the force increases, the indentation/brinelling intensifies, and the frictional force also increases to the point where there is no relative motion between the sample and the pin. Once this relative motion ceases, the friction-induced compliance error is minimized. Therefore, the swayback is only observed at low forces and does not affect the fracture toughness measurements. ASTM E1820 recommends removing the negative crack extension data if the reversal in the crack extension exceeds 0.5 mm[27]; however, the swayback in our present result is much less; therefore, we did not remove these data points from the *J*-R curve.

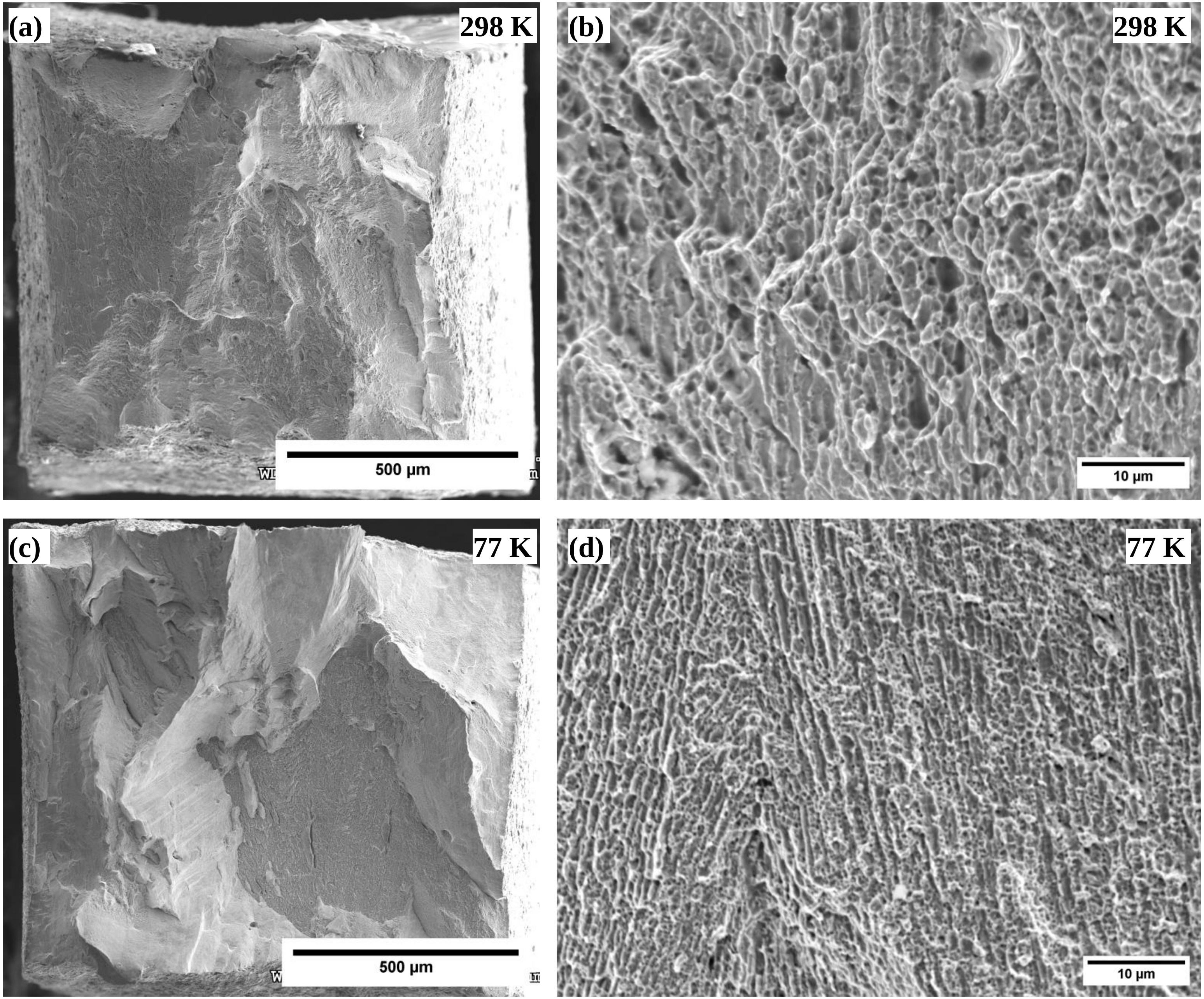

Supplementary Figure 13. The fractograph of the as-built microstructure tested at (a, b) 298 K, (c, d) 77 K. The specimens fail by the formation and coalescence of microvoids at both these temperatures.

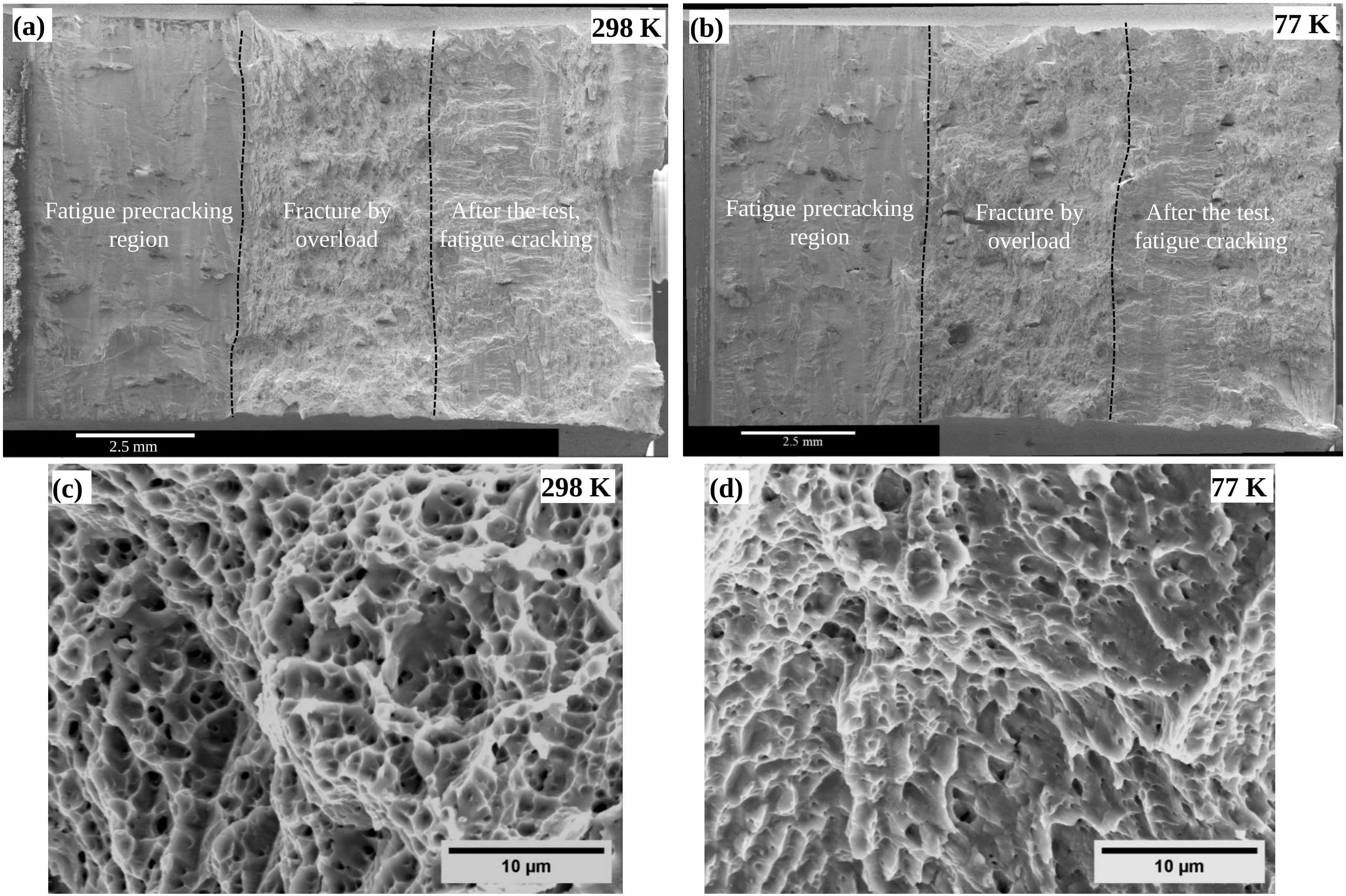

Supplementary Figure 14. The fractograph of as-built compact tension, C(T), specimens tested at (a) 298 K and (b) 77 K. The crack growth across the thickness is uniform, and the total crack growth length during fracture by overload (J-R evaluation) corresponds to the crack length measured by unloading complication measurements at the load line. Magnified image showing fracture by microvoid initiation and coalescence in specimens tested the (c) 298 K and (d) 77 K.

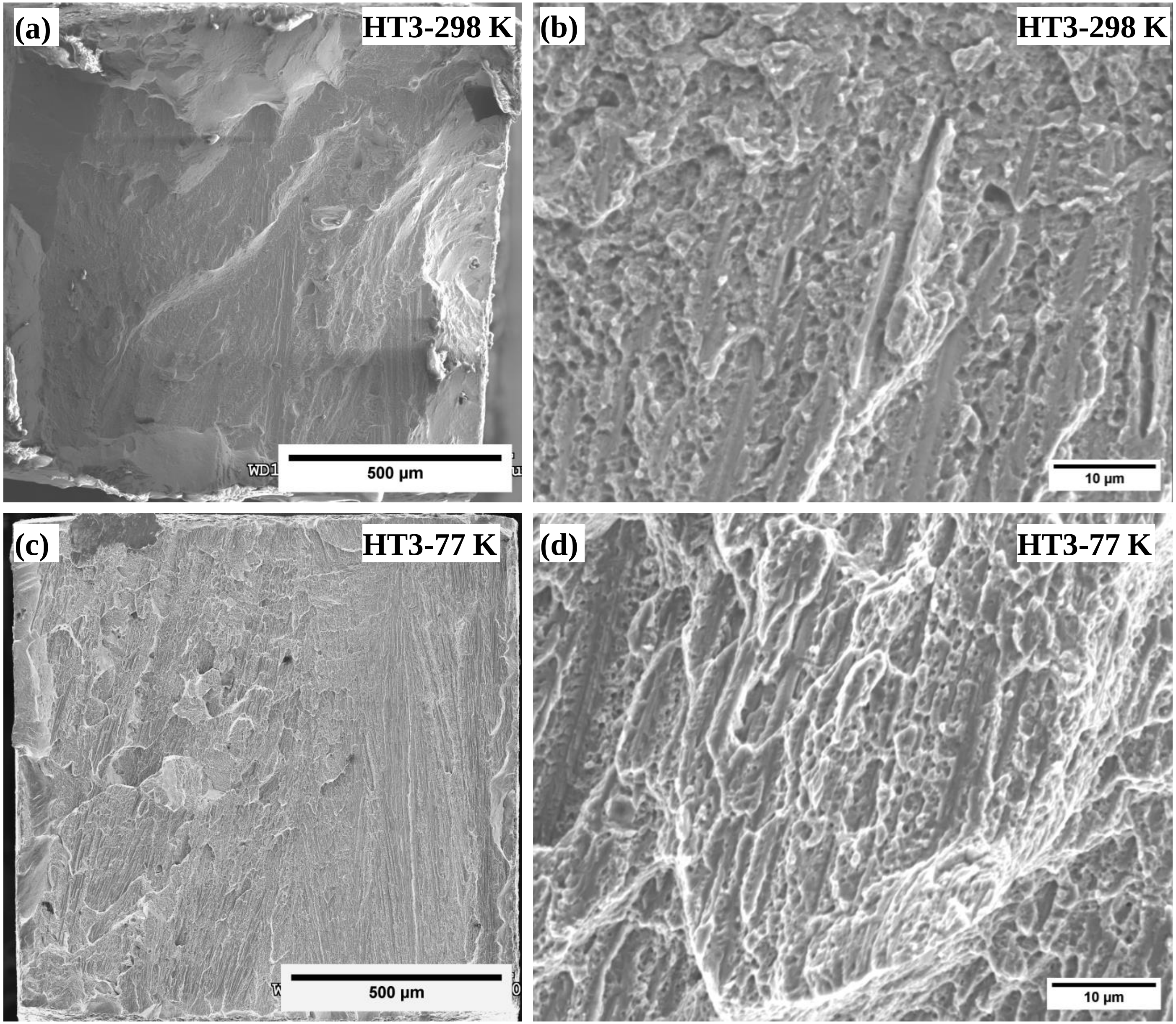

Supplementary Figure 15. The fractograph of the HT3 microstructure tested at (a, b) 298 K, (c, d) 77 K. The specimens fail by the formation and elongated microvoids of dimensions similar to the width of the honeycomb cellular structure.

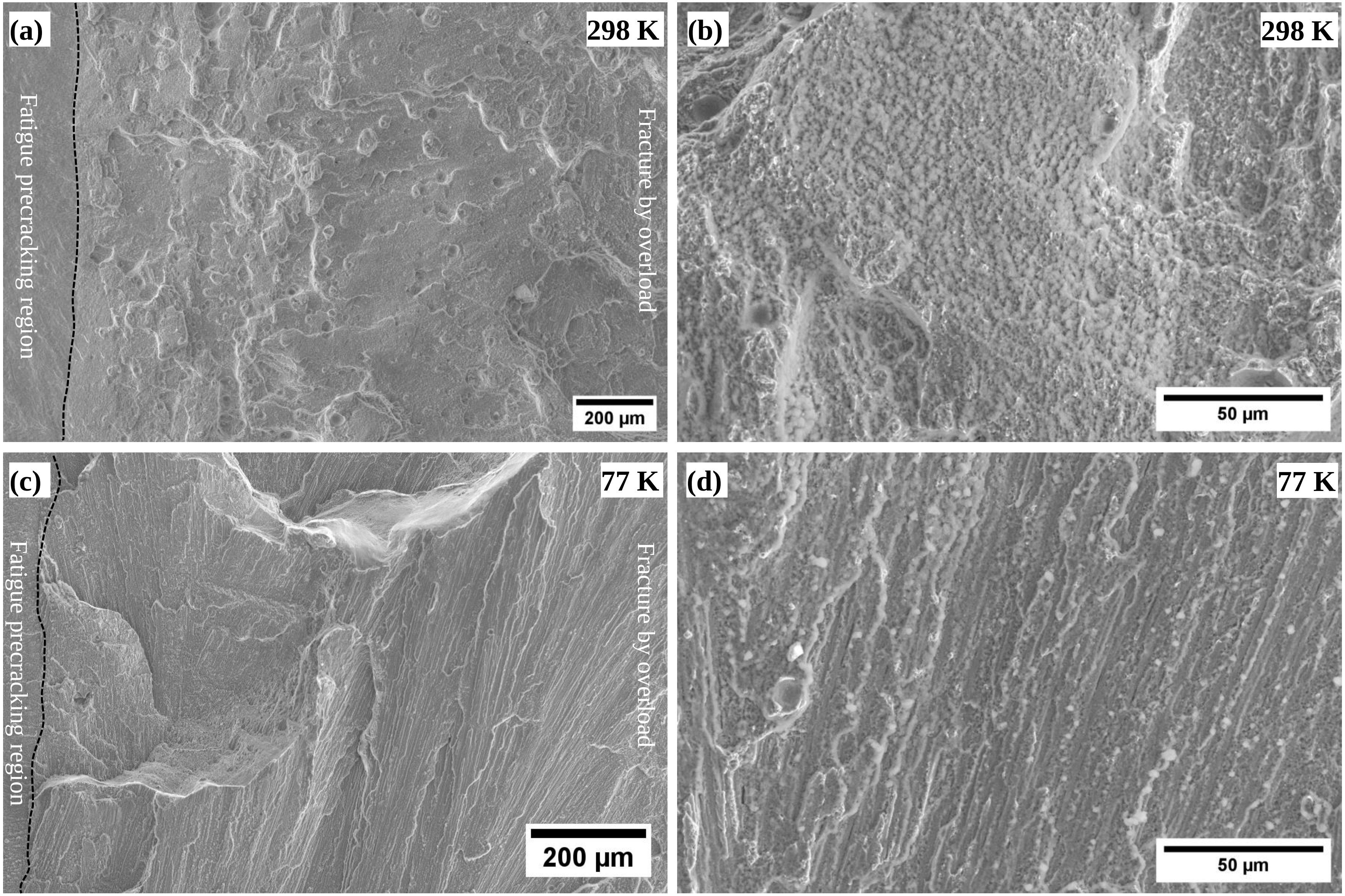

Supplementary Figure 16. The fractographs of HT3 compact-tension, C(T) specimens tested at (a, b) 298 K and (c, d) 77 K. The specimen was fractured by microvoid nucleation and coalescence at 298 K and cleavage-like fracture at 77 K.

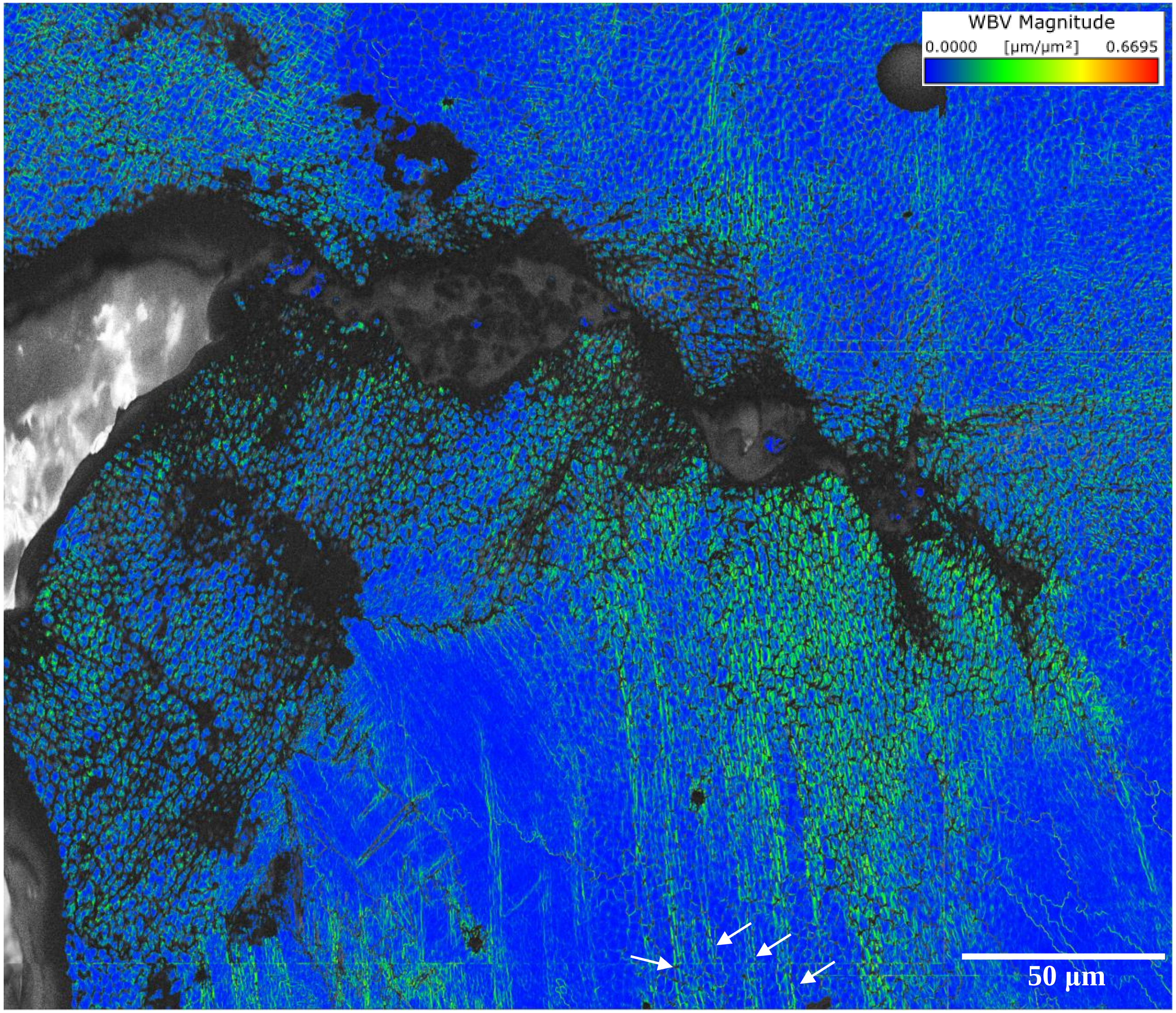

Supplementary Figure 17. The weighted burgers vector map showing dislocation bands (indicated by white arrows) that form the misorientation bands in the corresponding EBSD inverse pole figure (IPF) map in Figure 3.

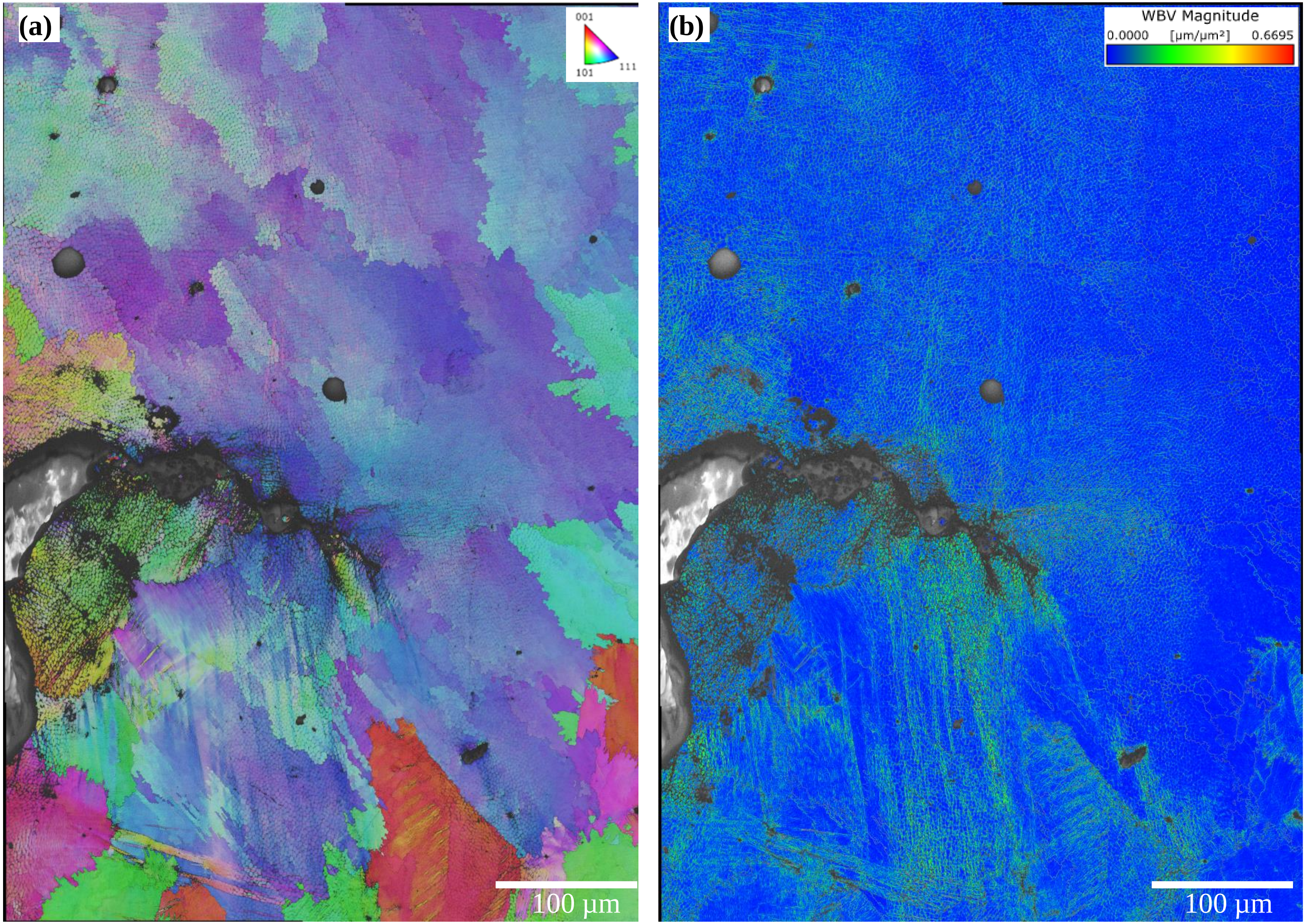

Supplementary Figure 18. (a, b) A larger area IPF map and corresponding weighted Burgers vector maps show the plasticity spread to a distance more than 300 μm away from the crack tip.

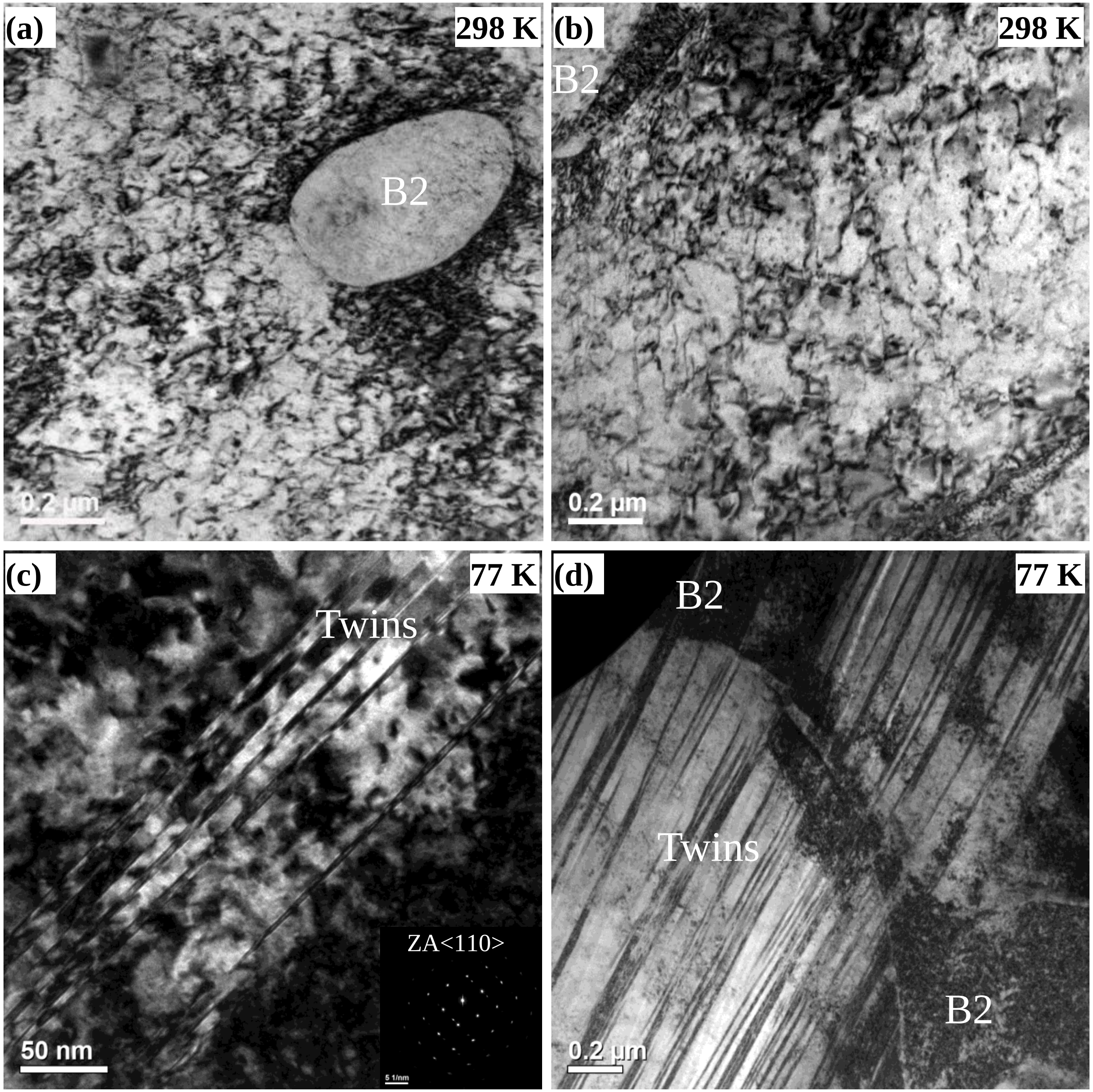

Supplementary Figure 19. Transmission electron microscopy images showing post-deformation microstructure inside the plastic zone of the C(T) specimen tested at (a, b) 298 K and (c, d) 77 K. The thick B2 precipitates on the cell boundaries restrict (a, b) the movement of dislocations at 298 K and (c, d) the growth of deformation nano-twins at 77 K.

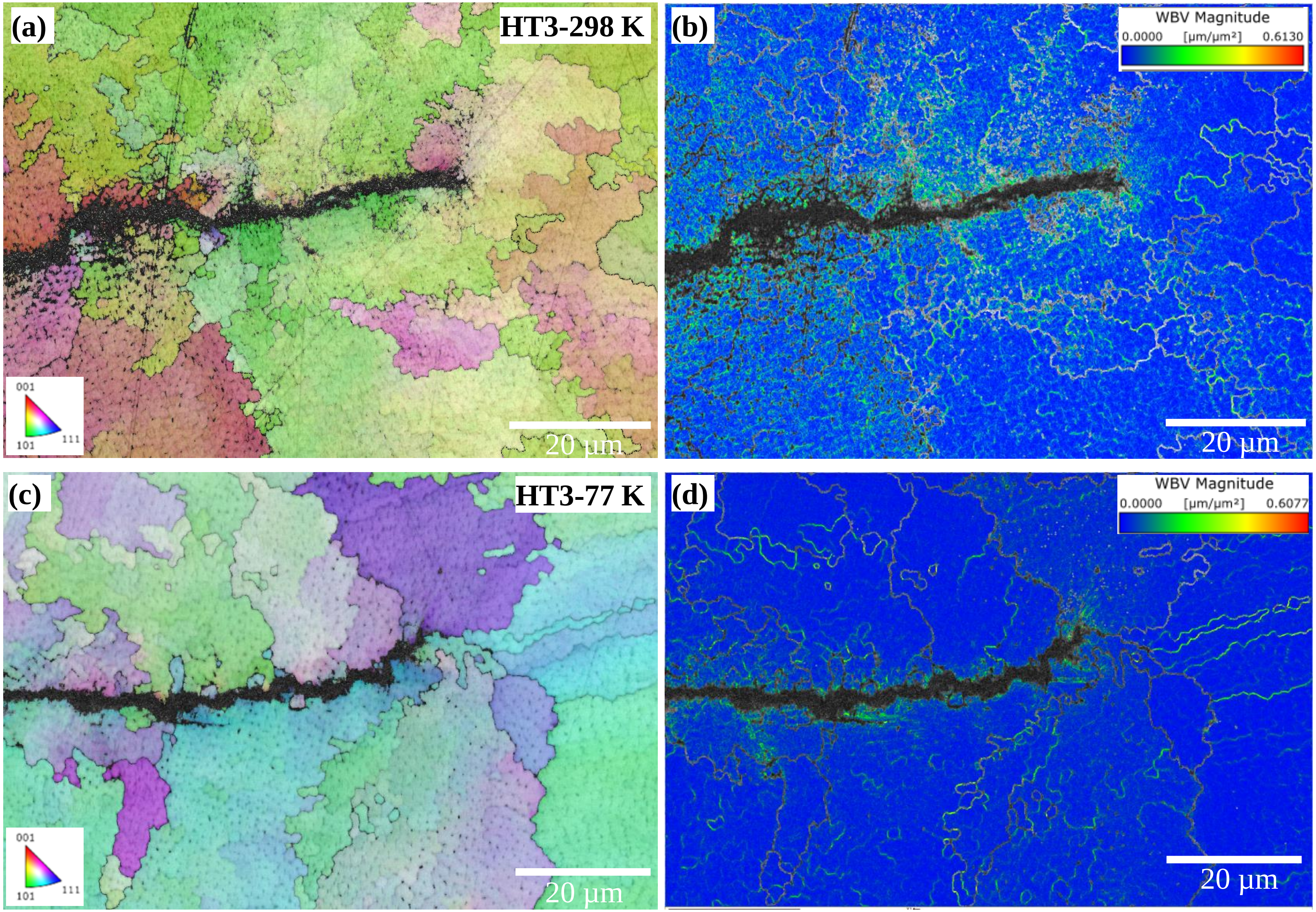

Supplementary Figure 20. (a, b) The EBSD IPF map and corresponding weighted Burgers vector map from the wake of the crack tip of the specimen fractured at 298 K after heat treatment HT3, the spread of plasticity is restricted to a maximum distance of 50 µm away from the crack tip. (c, d) The EBSD IPF map and corresponding weighted Burgers vector map from the wake of the crack tip of the specimen fractured at 77 K after heat treatment HT3, the spread of plasticity is restricted to a maximum distance of 10 µm away from the crack tip.

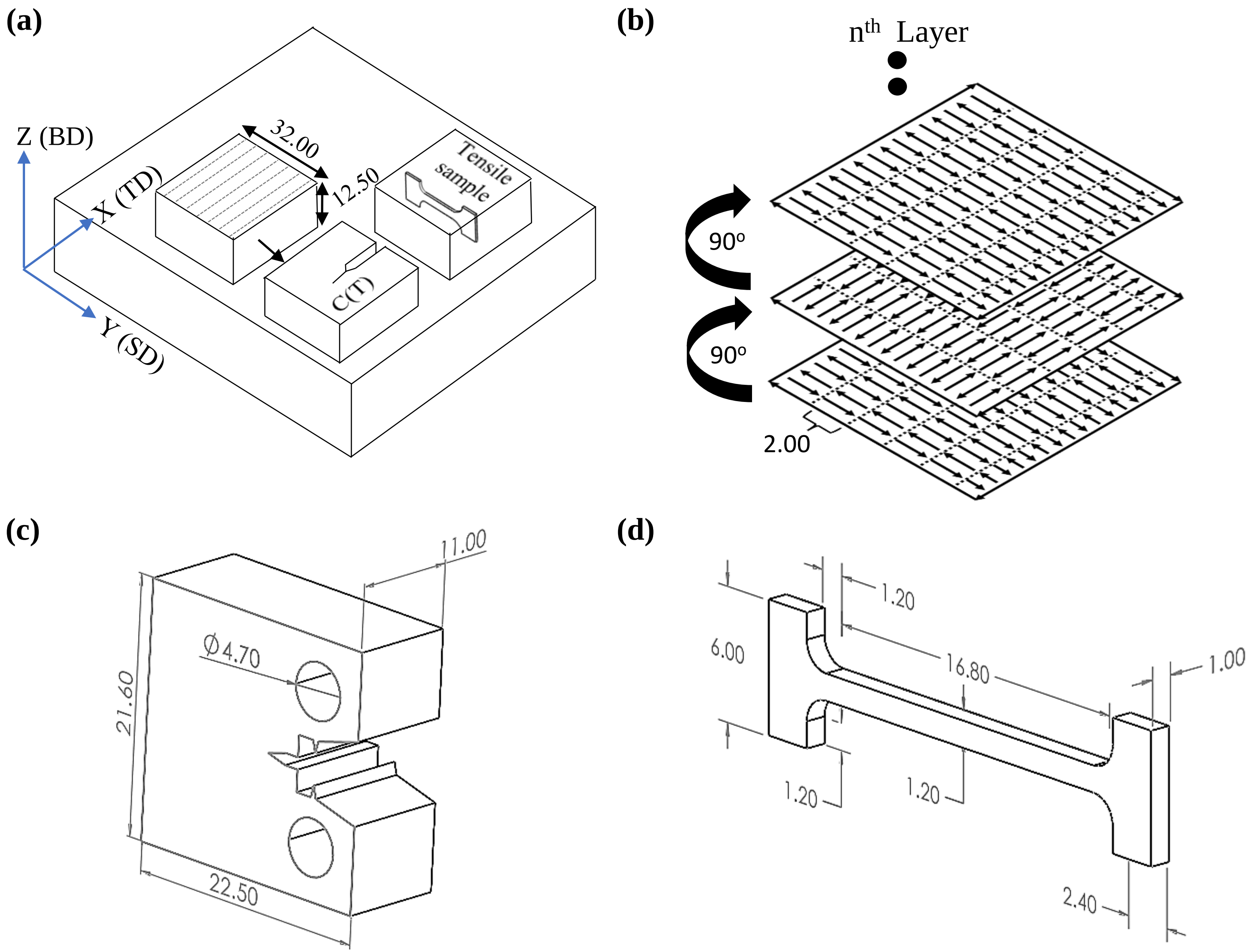

Supplementary Figure 21. (a) The relative orientation of the block printed with respect to the build plate and the orientations of compact-tension C(T) and tensile specimen machined from the printed blocks, where the X, Y, and Z-axes are parallel to the transverse direction (TD), scan direction (SD), and the build direction (BD). (b) Schematic illustration of bidirectional stripe scanning strategy for printing $Al0.5CrCoFeNi_5$ samples by L-PBF process. (c) Dimensions of C(T) sample and (d) dimensions of tensile specimens. All the dimensions are in mm.

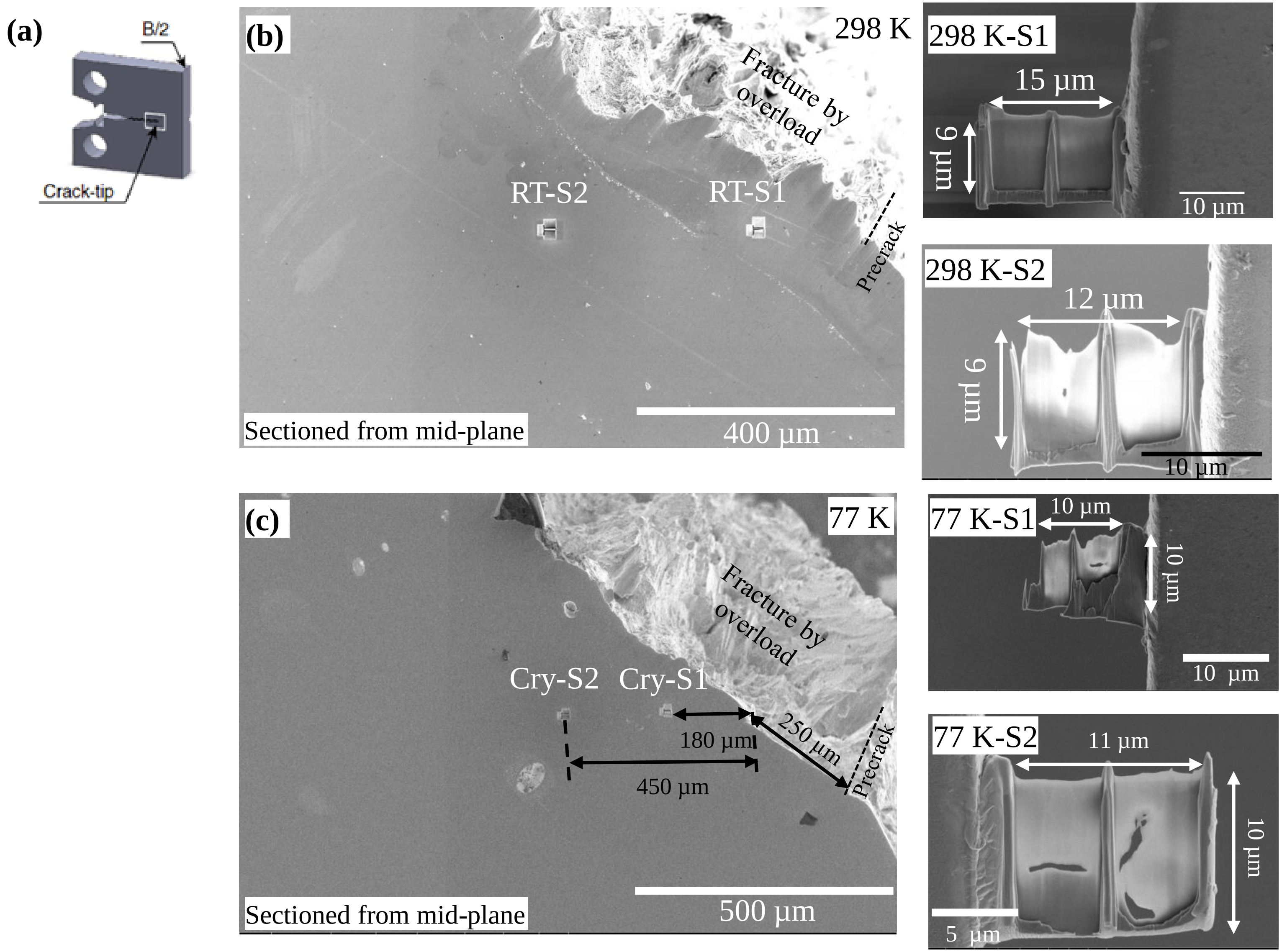

Supplementary Figure 22. (a) The schematic showing mid-plane slicing of the C(T) specimen to examine the microstructure at the crack tip in plane strain conditions. (b, c) The location of the focus ion beam (FIB) lifts out of the specimens for transmission electron microscopy

(a)

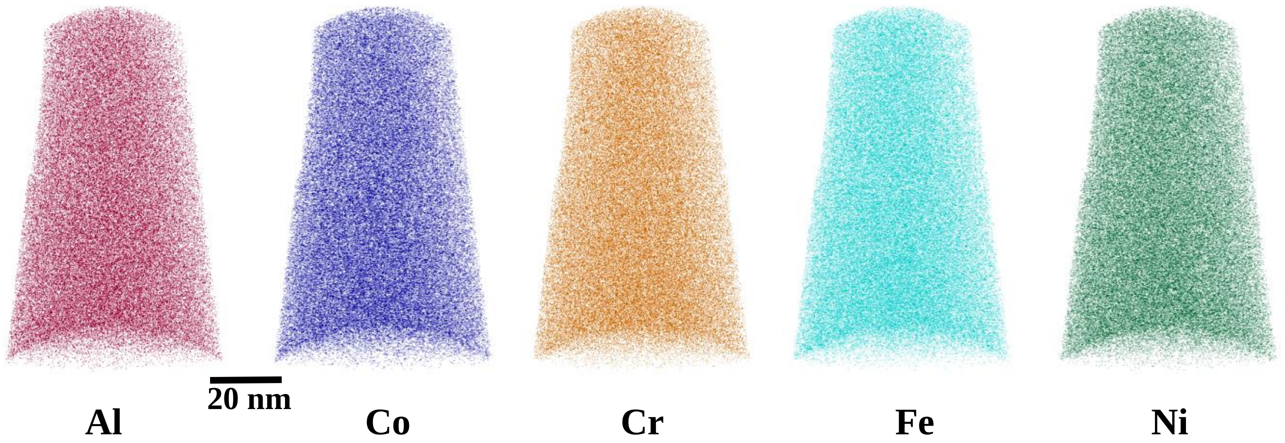

(b)

Elements	Composition (At.%)
**Cr**	21.43 ± 0.02
**Co**	22.35 ± 0.02
**Fe**	22.66 ± 0.021
**Al**	11.58 ± 0.015
**Ni**	21.97 ± 0.022

Supplementary Figure 23. (a) Atom probe tomography (APT) results of the interior of a *fcc* cell, (b) the average composition of the *fcc* phase matrix.
